# The Institutional Worker

**Published:** 1913-07-19

**Authors:** 


### INSTITUTIONAL WORKER

**Being a Special Supplement to "The Hospital"**

### OUR BUREAU OF INFORMATION.

### Rules for Correspondents.

1. Every letter must be accompanied by the coupon to be cut from the back cover (inside page) of The Hospital, current issue, and must contain the name and address of the correspondent with pseudonym for publication if desired. Replies by post cannot be given, save under exceptional circumstances at the Editor's discretion.

2. Letters from Approved Homes in reply to special needs published in the Bureau should state terms and full particulars, and be sent prepaid under cover to the Editor of the Bureau with name written across coupon for identification.

3. Proprietors of Homes which have not yet been entered on the List of Approved Homes, but have spare accommodation likely to suit special needs, are invited to write for an application form for registration. The fee for registration, which includes two announcements of the Home in the Bureau and other privileges, is 10s.

4. All communications to be addressed to the Editor of The Hospital, 28 Southampton Street, Strand, London, W.C., and marked "Bureau of Information."

### *SIDELIGHTS ON FINANCE.*

**Contributions from Institutional Officers under this heading are invited, and will be paid for in accordance with scale, if made use of.**

### An Alternative to Streets Collections.

A Hospital Saturday Secretary sends us the following account of his experience :—

Streets collections for charitable purposes have long been waning in popularity, and it is surprising, having regard to the disfavour with which they are viewed, that they are not replaced by a less objectionable method of collection. The truth is that the managers of many hospitals and other institutions would be glad to discard the system; but they hesitate to do so because they entertain misgivings as to the possibility of making good in some other way the loss of income which would thereby be entailed. The declining receipts from these collections, and the increasing difficulty in finding ladies willing to volunteer for this disagreeable work, are, however, strong indications of the need for a change. Such, at any rate, were the reasons which led our ladies' committee, after ineffectual attempts to popularise the collections, to adopt the bolder course of abandoning them altogether, and of formulating in their place a method of raising funds less likely to irritate their supporters and at the same time more congenial to the lady collectors.

The "Envelope" System.

After careful consideration our ladies' committee decided to adopt the "Envelope" system of house-to-house canvass, and the wisdom of their choice was evidenced by the excellent results achieved. The system is by no means new, for it has been practised for several years by some of the well-known charitable organisations in almost every town in the country, so that it cannot be said that its success depends now upon its novelty. On the contrary, the secret of success lies in the completeness of organisation and the enthusiasm of the workers. For the benefit of other institutions, I would here briefly outline the details of the scheme as handled by our ladies' committee.

Method of Organisation.

The town was mapped out into areas corresponding to the polling districts, each of which was allocated to a member of the ladies' committee, who undertook to preside over it and to provide a band of helpers to distribute and collect the envelopes. Each president was provided with a list of the streets included in her district and of the residents therein, prepared by cutting up the parliamentary register; in this way a canvass of every house in the town was arranged for without overlapping. Small cash bags, suitably inscribed, leaflets embodying a short appeal for contributions from the members of each family, and envelopes for enclosing them, also marked with an attractive inscription, were sent to the presidents for distribution to their collectors. The collectors delivered an envelope containing cash bag and leaflet to each house a few days before the appointed Hospital Saturday, and collected the cash bags with contents on that day, handing them to their respective presidents. Each lady collector was provided with a badge as an indication that she was duly authorised to collect. As an aid to the collectors, the date of the collection was kept well before the public by announcements in the local press, advertisements, and posters; indeed, the success of the scheme was dependent in no small degree upon this feature. Properly worked, the system produces better results than streets collections, and the work is far more congenial.

### How to Cheapen Insurance.

It is possible for hospital committees to insure their institutions at a reduction of 15 per cent. off the usual premiums charged. In other words, it means that the committee can, if it so elects, obtain the commission that would otherwise be payable to the agent. All that is needed is that the secretary or treasurer for the time being should be appointed as agent for the insurance company with which the insurance is effected, and the commission received handed over to the institution. This method is one largely adopted by our various Town Councils and other local authorities, and one that all the leading insurance offices are prepared to accede to. In those instances where special appliances are installed for extinguishing fires, special rebates are allowed in accordance with the nature of the installation.—"W. J. P."

### *INSTITUTIONAL FACTS AND FIGURES.*

### QUESTIONS FOR JULY.

**No. 1. (For the Officers of all Medical Institutions.)—State the character of your Institution, and describe your system of recording the admission, treatment, and discharge of patients admitted to the wards. Give descriptions of the various forms in use for the purpose, the method of dealing with them in the process of registration, and the method of filing history and treatment sheets. Add your further remarks.**

**No. 2. (For the Officers of Voluntary Hospitals.)—If you keep your accounts in accordance with the Uniform System, describe fully your method of checking, sorting, analysing, and filing your accounts for payment. Give such particulars as will clearly indicate the manner in which these are passed through your analysis journal, and onwards through the ledger. Add your further remarks.**

RULES.

The following rules must be observed:—

1. Contributions must be written on one side of the paper. They must bear the name and address of the sender and be accompanied by coupon to be cut from the back cover (inside page) of the current issue of The Hospital. A pseudonym must be chosen if the name is not to be published.

2. They must be addressed to the Editor of The Hospital, 28 & 29 Southampton Street, Strand, London, W.C., so as to reach him before the end of the month, and must be marked in the left-hand corner "Facts and Figures."

**A minimum payment of Half a Guinea will be made for each published answer.**

### *PRACTICAL MATTERS.*

### Electrical Apparatus.

The use of primary and secondary batteries for the electro-medical work of a hospital should be avoided if current from the town supply is available, for, unless they receive very careful attention, they quickly deteriorate, with the result that they are not available for use when required. The necessary supervision of electrical details is often lacking at the smaller institutions, and for this reason apparatus should be chosen which requires as little attention as possible. As current from the town supply is available at your institution, you will be well advised to purchase a transformer of the "earth-free" type. Several different designs have been placed on the market by the well-known manufacturers of electro - medical apparatus, amongst them the Multostat of the Sanitas Electrical Co., Ltd., is an excellent machine. These transformers, in addition to generating the various currents required for cautery, surgical lamps, electrolysis, etc., can be used as motors for driving surgical drills.—" N. D."

### Knife Cleaners.

Your complaint is a common one; many of the contrivances for cleaning knives, whilst perhaps they do their work well enough, certainly do shorten the lives of the knives. Try the "Acme Cutlery Renovator," made by the Avamore Engineering Company, of Kingsway, London; this machine cleans and polishes the entire blade in one operation without straining the handle, and protects the blade from undue wear.—" Cutlery."

### Bed-pan Cupboard.

A useful and well-ventilated bed-pan cupboard can be obtained of Messrs. Jas. Slater and Co., of the Holborn Engineering Works, London. It is made of iron, and is designed for fixing into the external wall of the sink-room, so that the greater portion of it protrudes on the outer side of the wall where the air has free access to the cupboard through the sides which are constructed in the form of weather-proof ventilators. The advantage of a cupboard of this sort for storing bed-pans over the practice of placing them on shelves in the sink-room is obvious. —" Sanitary."

### Ward Decoration.

There are so many excellent enamels on the market that it is scarcely possible to say which is the best. In our experience the durability and appearance of enamel depend almost as much upon the manner in which it is treated by the workmen as it does upon the quality, and for this reason we would suggest that you leave it to the decorators to specify a make that they are accustomed to work with. Though the initial outlay is greater, you will find that it is ultimately more economical to enamel your wards from top to bottom, including the ceilings; duresco for the upper part of the walls is favoured by some, but it retains the dust and will not stand a great deal of washing. Paint work properly varnished stands as well as enamel, though it does not present such a good appearance.—" Manager."

### *THE SICK AND IN NEED.*

**The Editor is prepared to make known without charge the needs of any who may be sick or in difficulties, and to guide them in making choice of Homes for treatment or convalescence. Reduced terms can often be arranged with Homes on the Approved List for those unable to pay full fees. Queries are specially invited from those who are engaged in any kind of philanthropical work. A new edition of the leaflet describing the purposes of the Bureau of Information is now ready and will be sent free of charge on application.**

### A Very Urgent Need.

Phthisis is responsible for a great deal of poverty and wretchedness, and this is an instance which merits much sympathy and all the help that can be rendered. We therefore appeal with confidence to our readers for their co-operation in assisting a family in a time of great need. The father is the victim of the disease, and he has been compelled, after repeated breakdowns, to relinquish his business career. He has a wife and two little children dependent upon him; his private means are exhausted, and his income is limited to the sickness benefit of 10s. weekly to which he is entitled under the National Insurance Act, and a small allowance in kind, in lieu of sanatorium treatment. The mother prior to her marriage was an elementary school teacher, and she could, were she able to get work, support herself and her children by teaching. There are several ways in which assistance might be rendered, as, for example, the employment of the mother in some suitable capacity whereby she may be enabled to support herself and her children, the provision of monetary assistance to tide over the present crisis, or the reception of the father into a suitable Home where he could be cared for free of charge. Co-operation can accomplish so much in a matter of this sort, and such help as our readers can give in one or other of the directions indicated will be gladly welcomed. The family is at present residing in the neighbourhood of Folkestone. Replies should be addressed " M. E. F.," 167b.

### *EMPLOYMENT AND TRAINING.*

### Qualifications of Queen's Nurses.

You cannot gain a three years' certificate in the way you suggest; training at one institution for three consecutive years is necessary. Three years' consecutive training, including at least two at an approved hospital or infirmary, is the minimum qualification for a Queen's nurse; where the training is divided between two institutions, the Institute usually requires that an arrangement be made between these institutions for the purpose. State the circumstances of your case to the Queen Victoria Jubilee Institute for Nurses, 120 Victoria Street, London, S.W., for their consideration. Having regard to the special circumstances, you might make application to the matron of your hospital to be allowed to complete your training.—" Anxious."

### Colonial Nurse's Prospects.

As a Colonial-trained nurse, there is no reason why you should not take up private nursing in this country, and you will, no doubt, find this branch of work most profitable. The possession of the C.M.B. certificate should help you considerably. The fees for general nursing range from a guinea and a half a week, and for midwifery from two guineas. Colonial nurses are accepted by some private nursing institutions, though they are excluded from the most important. A three-years' training at an approved general hospital or infirmary, either in this country or the Colonies, is the primary qualification for a Queen's nurse, and nurses so qualified can receive the necessary district training under an agreement with the Queen Victoria's Jubilee Institute or one of the affiliated Homes approved by the Institute. If you desire to take up this work, you should apply to the General Superintendent at 120 Victoria Street, London, S.W., stating the name of your training school. A stamped envelope should be enclosed for a reply.—" M. C."

### EDITOR'S LETTER-BOX.

*M. P.—Case of Epilepsy.*—We are glad to learn that some one has been found willing to undertake the supervision of this case, and we trust that a satisfactory arrangement will be completed. We are always pleased to know that the efforts of the Bureau are appreciated.

*B. Arnold.*—The last communication received from you was acknowledged in these columns on April 19. We are sorry to hear that you have received no replies to your letters; we always request that this courtesy be extended to the principals of Homes, but we are unable to enforce it. An advertisement may help you as you suggest, and we are handing your letter to the business manager.

*M. E. F. — Case of Advanced Phthisis.*—We should be glad to hear whether this case was eligible for St. Michael's Home, Uxbridge. We are still making efforts to assist the family.

*Wayne.*—We were glad to have your letter, and trust that the Home, the particulars of which we forwarded you, will suit. Should you not be successful in coming to a satisfactory arrangement, you might apply to the Superintendent, Princess Marie Louise House, Y.M.C.A., Worthing.

*The Editor is indebted for testimonials in response to inquiries from* M. B. Browne and L. Semple.

### Editor's Notices.

**Contributions:**

Contributions should be written, or preferably typed, on one side of the paper only, and all articles sent in are accepted upon the distinct understanding that they are forwarded to The Hospital only.

The Editor cannot undertake to return MSS. not used, but when a stamped directed envelope is enclosed the MSS. may be returned if a special request is made.

Accepted articles and paragraphs of news will be paid for after publication at the scale rate.

**Address:**

To prevent delay all contributions and letters on editorial business must be addressed exclusively to the Editor, "The Hospital," 29 Southampton Street, Strand, London, W.C. It is important that this regulation shall be strictly observed.

**Correspondence:**

Correspondence on all subjects is invited. The name and address of correspondents must be given as a guarantee of good faith, but not necessarily for publication.

**Special Articles:**

Special articles are invited, and questions, inquiries, and paragraphs upon all matters relating to the work, administration and management of general, special, mental, fever, cottage and convalescent hospitals, sanatoria, homes, institutions, societies and organisations for the treatment or care of the sick, injured and dependants of all classes. Every matter affecting the interests and welfare of the staff of all grades working in these institutions will receive special consideration.

**Administrative Medicine, Research and Items of News:**

Special terms will be paid for approved articles on subjects in this wide field by experts who have made some section of it their special study and interest. We wish to give prominence to every movement tending to advance accuracy of diagnosis, efficiency in treatment, and the development of modern methods to eradicate disease and promote the welfare of the suffering.

**A Bureau of Information:**

We invite inquiries and applications for information and help in providing for that numerous class of sufferers who require special care or house-room which they cannot obtain in their own homes or secure for themselves. We cannot, however, prescribe, or recommend practitioners.

**Books for Review:**

Publishers are particularly requested to send advance proofs of any new books of importance whenever possible, as the Editor has made arrangements to publish immediate reviews on a new plan.

**Photographs, Plans, Blocks, and Illustrations:**

It is requested that wherever possible MSS. may be accompanied by illustrations in any of the above forms. The name of the sender and of the article to which it belongs should be written on the back of each photograph, drawing, or block for purposes of identification.

**Local Papers:**

Newspapers containing reports or news paragraphs should be marked and addressed to the Sub-Editor.

**The Coupon System:**

A coupon will be found at the bottom of the third inside page of the cover each week. This coupon must be attached to every question or inquiry to which an answer is desired in The Hospital.

### A Great Help.

In consequence of the ever-increasing circulation of The Hospital it is often a matter of considerable difficulty to obtain a copy of the current issue at the local newsagents. In these circumstances we strongly recommend you to guard against the contingency of The Hospital being sold out by sending a subscription to the Journal either through a bookseller or direct to the Publishing Offices. By this means it is possible to secure a copy of the Journal almost immediately upon publication.

This is *a great help* when seeking a post, as it enables you to send in your application without delay, an advantage which may secure you the appointment. In addition to this, as a subscriber, no matter how many times you may change your address, or in what part of the country you may be at the moment, a postcard will ensure that you receive The Hospital on the same day and at the same hour as when at home.

**Subscriptions** may begin at any time, and are payable in advance. Cheques and Post-Office Orders should be crossed London County and Westminster Bank, Covent Garden Branch, and made payable to the Manager of The Hospital.

Rates of Subscription (payable in advance).

| | | |
|---|---|---|
| **UNITED KINGDOM.** | | |
| Three Months (including Postage) | ... ... ... ... | 2s. 0d. |
| Six Months | do. ... ... ... ... | 4s. 0d. |
| Twelve Months | do. ... ... ... ... | 6s. 6d. |
| **FOREIGN AND COLONIAL.** | | |
| Three Months (including Postage) | ... ... ... ... | 2s. 6d. |
| Six Months | do. ... ... ... ... | 5s. 0d. |
| Twelve Months | do. ... ... ... ... | 8s. 8d. |

### *SUBSCRIPTION FORM.*

*Please send me* The Hospital *for..............months, commencing with your issue of.............................., for which please find enclosed............................*

*Name*.........................................................

*Address* .........................................................
.........................................................

*Date*....................................

*To* .................................... *Newsagent.*

### Manager's Notices.

**Letters relating to the publication, sale, and advertising departments of "The Hospital" must be addressed to The Manager, "The Hospital," The Hospital Building, 28 and 29 Southampton Street, London, W.C.**

**Advertisements:**

To ensure insertion, all advertisements for The Hospital must reach the Manager not later than Wednesday morning in each week. For scale of charges see pages v and 4.

**Remittances:**

**It is especially requested that remittances be made by Cheque or Postal Order, and in halfpenny stamps only when the amount is under sixpence.**

### HANDBOOKS
### For TRAINED WORKERS

POCKET GUIDE SP SERIES

**1/- net. 1/1 Post free.**

**Bound in Limp Cloth.**
**Suitable Size for the Pocket.**

---

**The works which comprise the S.P. Pocket Guide Series are intended for ready reference, consequently the aim of the Publishers has been to make them as concise and lucid as possible. Each book is written by an expert on the subject of which it treats, and the utmost reliance therefore can be placed in the information it imparts.**

---

**Essentials of Fever Nursing.** By LYTTON MAITLAND, M.D. (Lond.), M.B., B.S., D.P.H. (Camb.).

**Manual and Atlas of Swedish Exercises.** (*With over 60 Illustrations.*) By THOMAS D. LUKE, M.D., F.R.C.S.

**Bandaging Made Easy.** (*Illustrated with over 90 Diagrams.*) By M. HOSKING, Sister-in-Charge, Tredegar House, Bow, E.

**How to Write and Read Prescriptions.** By LYTTON MAITLAND, M.D. (Lond.), M.B., B.S., D.P.H. (Camb.).

**Principal Drugs and their Uses.** By A PHARMACIST.

**Asepsis and How to Secure It.** By H. W. CARSON, F.R.C.S.

**Treatment after Operations.** *Rules for Nursing after General and Special Operations.* By MARY WILES.

**The Nurse's Duties before and during Operations.** By MARGARET FOX, Matron, Prince of Wales's General Hospital, Tottenham.

**Other works, in amplification of this Series, will be announced in due course.**

---

***Published by***

### The SCIENTIFIC PRESS, Ltd.

**28/29 Southampton Street, STRAND, LONDON, W.C.**

NEARLY

**400 NEW WORDS**

have been added to the

**NEW EDITION**

of

### The Nurse's Pronouncing Dictionary

**OF MEDICAL TERMS AND NURSING TREATMENT**

Compiled by **HONNOR MORTEN.**

Eighth Edition, thoroughly Revised and much Enlarged.

Edited by **MARY I. BURDETT.**

The new issue of this well-known Dictionary is the **most complete work** of the kind published. It has been completely revised and some hundreds of new words have been added, consequently the utmost reliance can be placed in the information it contains.

To be up-to-date you should GET A COPY TO-DAY.

**Price, in cloth, 2/- post free; in leather, 2/6 net, by post 2/8.**

*Of all Booksellers, or of—*

**The Scientific Press, Ld.**

(*The Nurses' Publishers*),

**28/29 Southampton Street, STRAND, LONDON, W.C.**

